# The impact of a partnership care model on self-efficacy and self-care in hemodialysis patients: A quasi-experimental study

**DOI:** 10.1016/j.conctc.2025.101459

**Published:** 2025-02-27

**Authors:** Khodayar Oshvandi, Hossein Moradi, Salman Khazaei, Azim Azizi

**Affiliations:** a Mother and Child Care Research Center, Institute of Health Sciences and Technologies, Hamadan University of Medical Sciences, Hamadan, Iran; b Department of Medical Surgical Nursing, School of Nursing and Midwifery, Hamadan University of Medical Sciences, Hamadan, Iran; c Research Center for Health Sciences, Institute of Health Sciences and Technologies, Hamadan University of Medical Sciences, Hamadan, Iran; d Department of Epidemiology, School of Public Health, Hamadan University of Medical Sciences, Hamadan, Iran; e Chronic Diseases (Home Care) Research Center, Institute of Cancer, Hamadan University of Medical Sciences, Hamadan, Iran

**Keywords:** Self-care, Self-efficacy, Partnership care model, Hemodialysis, End-stage renal disease

## Abstract

**Introduction and objective:**

Self-care and self-efficacy are crucial in managing kidney failure requiring hemodialysis. However, traditional education methods have not effectively improved self-care and self-efficacy. Therefore, utilizing more effective models, such as the partnership care model, is essential. This study aimed to determine the impact of the partnership care model on self-care and self-efficacy in hemodialysis patients.

**Materials and methods:**

This quasi-experimental study was conducted with 74 hemodialysis patients at Shahid Beheshti Hospital in Hamadan. Patients were divided into control and intervention groups. The control group received only routine care, while the intervention group received nursing care based on the partnership care model in addition to routine care. This included eight educational sessions over one month, with two sessions per week. Data were collected using self-care and Sherer self-efficacy questionnaires before and two months after the intervention. Data were analyzed using SPSS version 16.

**Results:**

The study demonstrated significant improvements in self-care and self-efficacy among hemodialysis patients following the collaborative care intervention. Post-intervention, the experimental group showed substantial benefits with an effect size of 1.41 for self-care (95 % CI: 0.87–1.95, p < 0.001) and an effect size of 1.55 for self-efficacy (95 % CI: 0.99–2.10, p < 0.001), highlighting the effectiveness of the intervention.

**Conclusion:**

The study demonstrated that the partnership care model, through comprehensive education and social and motivational support, significantly improved self-care and self-efficacy in hemodialysis patients. Therefore, it is recommended that hospitals and nurses adopt this model to enhance the health and quality of life of patients.

## Highlights

• The partnership care model boosted self-efficacy & self-care.
• Training nurses in this model is key for effective implementation.

**Summary Box**

**What is already known about the topic?**

• Routine care and education can lead to improvements in patient self-care.
• Partnership care model have been shown to improve self-efficacy and self-care in various patient populations.

**What does this study add?**

• This study demonstrates that a comprehensive, multi-stage partnership care model significantly enhances self-efficacy and self-care in hemodialysis patients.
• The study identifies key factors and mechanisms, such as increased knowledge, motivation, social support, and active patient participation that contribute to these improvements.

**Clinical implications**.

• Implementing partnership care model that include stages of motivation, preparation, engagement, and evaluation can effectively improve self-care and self-efficacy in hemodialysis patients.
• Training nurses to use this model can enhance patient outcomes and support the integration of collaborative care into routine clinical practice.

## 1 Introduction

Chronic kidney disease (CKD), primarily caused by diabetes and hypertension, had a global prevalence of 9.1 % in 2017, affecting approximately 700 million people [1]. The incidence of end-stage kidney disease (ESKD), treated with dialysis and kidney transplantation, increased by 43.1 % and 34.4 %, respectively [2]. Mortality due to CKD has risen by 41.5 % since 1990, making CKD the 12th leading cause of death worldwide in 2017 [3]. Patients with ESKD require renal replacement therapies such as hemodialysis, peritoneal dialysis, or kidney transplantation to survive [1].

Patients undergoing hemodialysis face complications such as hypotension, muscle cramps, nausea and vomiting, headaches, chest pain, itching, fever, and chills [4,5]. Due to multiple medication regimens, specific dietary plans, and the need to adapt to physical and psychological disabilities, as well as disease-related complications, these patients require self-care activities [[6], [7], [8], [9]]. Self-care involves performing certain physical care aspects and actively participating in one's care, ultimately reducing healthcare costs [10]. In the realm of hemodialysis, self-care practices include adhering to the dialysis schedule, following dietary and fluid restrictions, and managing symptoms and complications [11].

Self-efficacy, a key concept in Bandura's social cognitive theory, is crucial for improving patient health. Self-efficacy refers to the belief in one's ability to successfully execute a specific behavior and expect the resulting outcomes. It is a critical prerequisite for behavior. Bandura posits that creating an environment conducive to acquiring necessary skills and knowledge and achieving success can enhance self-efficacy and empowerment. According to his theory, an individual's perception of their ability leads to the adoption of self-care behaviors to achieve desired outcomes [12,13]. Evidence suggests that increased self-efficacy in dialysis patients improves weight control between dialysis sessions, reduces hospitalizations, decreases amputations, and enhances quality of life, particularly in diabetic dialysis patients [14]. Additionally, increased self-efficacy promotes behavior change, treatment adherence, and overall physical and mental health improvement [15].

The existing literature indicates that self-care and self-efficacy in hemodialysis patients are low, necessitating effective methods to enhance them [[16], [17], [18]]. Hosseini et al. (2022) found that many hemodialysis patients had low levels of self-care and self-efficacy before intervention which negatively impacted their treatment outcomes. This finding underscores the need for effective interventions to enhance self-care and self-efficacy among these patients [19].

Modern nursing care emphasizes using nursing models to develop patient education. One such model is the partnership care model, first designed by Mohammadi and colleagues in 2002 and implemented and evaluated for hypertension control in Babol [20]. In the partnership care model, the quality and type of relationship between the parties in the care process are crucial, with the patient's participatory role in treatment and care being essential [21]. Additionally, employing a culturally appropriate model plays a significant role in improving individual health [20].

Participatory care is a systematic process for establishing effective, interactive, and continuous communication between the patient, an active family member, the center's physician, and the nurse as the healthcare provider. This process aims to identify needs and problems, sensitize patients to adopt continuous health behaviors, and help maintain recovery and health improvement. Its steps include motivation, preparation, engagement, and evaluation [22]. Various studies have shown the impact of the partnership care model on reducing fatigue severity in hemodialysis patients [23], improving symptoms in patients with depression [24], reducing depression levels in adolescents with major thalassemia [25], and enhancing self-care, quality of life, and cardiac function in patients with CHF [26].

Managing treatment poses significant challenges for dialysis patients, making the implementation of effective care models crucial. Although previous studies have demonstrated that nursing care models can enhance the self-efficacy and self-care of dialysis patients [6,7,27,28], introducing the collaborative care model presents a novel approach to further improving these outcomes. In this model, patients are acknowledged as active partners in their treatment process, which can bolster their sense of responsibility and heighten their motivation to effectively manage their health. This unique aspect has the potential to make this model more effective in disease management compared to other nursing care models [20]. Additionally, education using locally and culturally appropriate nursing models may have a greater impact on enhancing patient outcomes [20]. The partnership care model, which emphasizes patient participation and has shown positive outcomes in other conditions such as hypertension, depression, and heart failure [[23], [24], [25], [26]], has not yet been applied to enhancing self-care and self-efficacy in hemodialysis patients. This represents an unexplored area in the literature. Our study specifically addresses this gap by evaluating the effectiveness of the partnership care model in improving self-efficacy and self-care among hemodialysis patients. This study aimed to determine the impact of the partnership care model on self-efficacy and self-care in hemodialysis patients.

## 2 Methods

### 2.1 Type of study

This quasi-experimental study, involving both a control and an experimental group, was conducted before and after the intervention. Participants included male and female hemodialysis patients from the hemodialysis units at Shahid Beheshti Hospital in Hamadan, who met the inclusion criteria. The research process commenced in November 2023 and concluded at the end of September 2024. It is important to acknowledge that blinding was not implemented in this study, resulting in an open-label design. As a result of the lack of blinding, two potential biases, expectation bias and response bias, may occur, which are partly beyond the control of researchers.

### 2.2 Sample size and calculation method

According to the findings of the 2017 study by Hua et al. [26], the self-efficacy scores following the intervention were approximately 58 for the collaborative care group and 53 for the control group, with a standard deviation of 7 in both groups [26]. To achieve a statistical power of 80 % while maintaining an alpha level of 0.05 within a 95 % confidence interval, the required sample size was calculated to be 31 participants per group. After accounting for an anticipated attrition rate of 20 %, the adjusted final sample size for each group was set at 37 participants.

$$\eta =\frac{{\left(Z_{1-\frac{\alpha }{2}}+Z_{1-\beta }\right)}^{2}\left(\sigma_{1}^{2}+\sigma_{2}^{2}\right)}{{\left(\mu_{1}-\mu_{2}\right)}^{2}}$$

**Figure:**
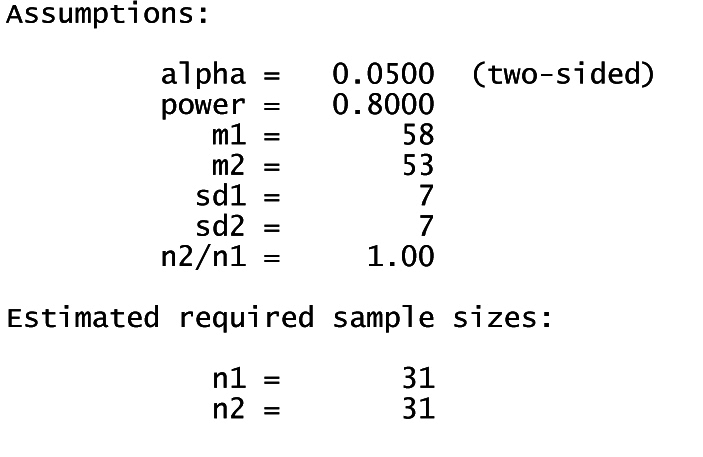

### 2.3 Sampling method

To prevent selection bias between the study groups, patients were divided based on their visit days to the male and female hemodialysis units into two categories: those visiting on even days and those visiting on odd days. Using a lottery method, odd days were assigned to the control group and even days to the test group. From the patients visiting on odd or even days, research samples were selected using simple random sampling. Lists of patients visiting each unit on even and odd days were prepared separately, and research samples were selected using random numbers generated by software. Patients meeting the inclusion criteria were included in the study.

### 2.4 Inclusion and exclusion criteria

Inclusion criteria were: at least six months since the start of dialysis and a stable final diagnosis, undergoing hemodialysis two to three times a week, aged between 18 and 70 years, and able to understand Persian. Exclusion criteria included missing more than one training session, unwillingness to continue participation, worsening of the disease to the extent that the patient could not communicate, migration, or death.

### 2.5 Data collection tools and validity and reliability of the tools

Data collection tools included a demographic and clinical information form, the Baghyani Moghadam Hemodialysis Patients' Self-Care Questionnaire, and the Sherer Self-Efficacy Scale.

#### 2.5.1 Demographic information form

The demographic information form included personal details such as sex, marital status, occupation, education, number of dialysis sessions per week, age, duration of kidney problem, duration of dialysis, body mass index, duration of each dialysis session, and monthly household income.

#### 2.5.2 BaghianI moghadam hemodialysis patients' self-care questionnaire

This tool consists of 18 items, each rated on a 5-point Likert scale from “never" (0) to “always" (4). Higher scores indicate higher levels of self-care ability. This questionnaire is unidimensional [29]. In the study conducted by BaghianI Moghadam et al. (2016), the validity and reliability of this tool were determined through item clarity assessment. A questionnaire was administered to 10 hemodialysis patients, who were asked to respond to the questions and raise any inquiries regarding the items. Based on the feedback received from these individuals, item clarity was established. To assess the face and content validity of the questionnaire, six copies were distributed to a panel consisting of three health education specialists, one nephrologist, one dialysis nurse, and one statistics expert, all of whom approved the questionnaire. For internal reliability assessment, a preliminary study was conducted with a sample size of 15 participants, resulting in Cronbach's alpha coefficients of 0.88 and 0.84 for the self-care, barriers, and benefits items, respectively [29]. In this study, the reliability of the self-care questionnaire designed for hemodialysis patients was evaluated through its administration to a sample of 15 individuals undergoing hemodialysis. Following the completion of the questionnaire, the Cronbach's alpha coefficient was calculated, yielding a value of 0.90, indicating a high level of internal consistency.

#### 2.5.3 Sherer Self-Efficacy Scale

This questionnaire was developed by Sherer et al., in 1982 and translated into Persian by Zare et al. It consists of 17 items and is unidimensional. The items are rated on a 5-point Likert scale from “strongly disagree" (1) to “strongly agree" (5). Items 2, 4, 5, 6, 7, 10, 11, 12, and 14 are reverse scored. Higher scores indicate higher self-efficacy [30,31]. In assessing the validity of the General Self-Efficacy Scale constructs, the scores obtained from this scale were correlated with measures of several personality traits. The predicted correlation between the self-efficacy scale and the personality trait measures was 0.61, which was statistically significant at the 0.05 level, supporting the construct validity of the intervention in question [30]. The reliability coefficient of the scale, determined using the split-half method according to Guttman, was found to be 0.76, while the Cronbach's alpha coefficient was 0.79 [32]. In Iran, a study conducted by Barati aimed at evaluating the validity and reliability of this scale reported a correlation of 0.61 between the self-esteem and self-assessment scales and the self-efficacy scale, further supporting the construct validity of this scale [31]. The reliability of the questionnaire, assessed using Cronbach's alpha, was found to be 0.86 in the study by Noghani et al. [33]. The reliability of the self-efficacy questionnaire was assessed with 15 hemodialysis patients, resulting in a Cronbach's alpha of 0.88, indicating high internal consistency.

### 2.6 Data collection method

After selecting the samples, the demographic and clinical variables form and the self-care and self-efficacy questionnaires were completed by patients in both groups through self-reporting. For illiterate patients, structured interviews were conducted using these questionnaires by the patients' companions.

#### 2.6.1 Control group

The control group received routine care and education from the nurses. Routine hemodialysis care in Iran includes pre-dialysis preparation, ensuring both the dialysis machine and patient are ready for the session. During dialysis, vital signs like blood pressure and heart rate are monitored, and any arising complications are managed. Post-dialysis care involves checking for bleeding, controlling blood pressure, and recording weight changes. Infection control practices are strictly followed to prevent infections. Patients undergo monthly comprehensive dialysis assessments and weekly specialist nephrology visits. Hepatitis B vaccinations are administered when needed. Regular medication management and nutritional guidance are provided. Care for the vascular access site is emphasized to prevent complications. Emotional and social support are offered to both patients and their families.

#### 2.6.2 Intervention group

The intervention group sessions were conducted in 8 sessions with the patient, an active family member, and a nurse, each lasting 45–60 min at the patient's bedside during hemodialysis. The education sessions began during the preparation stage and before the start of dialysis. At this stage, patients are more refreshed and alert, allowing them to better focus on the information and education provided. The educational programs were based on the steps and stages of partnership care model, including motivation, preparation, engagement, and evaluation [20], implemented over 8 sessions in one month. Each week, two 45–60 min sessions were held at the patient's bedside during dialysis.

*Motivation Stage****:*** In the motivation stage, actions were taken to motivate the patients (patient and family member) based on the principles and goals of the model. Efforts were made to make patients and family members aware of the importance of self-care and their active role in managing the disease. This was done through educational sessions and individual counseling, explaining how simple actions like following a proper diet and regular treatment could improve self-care. Success stories of other patients were also used to boost motivation and hope. Additionally, patients and family members were familiarized with care problems, current conditions, threats, and complications of the disease, and psychological and physical symptoms. The content of these sessions included a review of kidney anatomy, causes of kidney failure, physical and psychological problems resulting from it, aggravating and alleviating factors. Discussions were held about dialysis and its types, complications, and prevalence with patients and their families. The consequences of not following nursing and medical recommendations were explained. The timing of subsequent sessions was coordinated with them. This stage was conducted in two 45–60 min sessions during the first week for all test group members individually and face-to-face.

*Preparation Stage****:*** Visits were conducted to implement the second stage, preparation of patients for participation in the care and treatment process. After this session, patients should be able to explain physical and psychological symptoms and methods to reduce these complications and problems. Patients should list their daily tasks and perform them according to the plan. The content of these sessions included teaching methods to organize daily life activities, such as daily planning and listing daily tasks. The goal was to prevent problems that exacerbate physical and psychological complications. Sessions were held with the presence of a nurse, patient, and active family member, involving them in decision-making and the presentation of sessions and educational content. Education in these sessions was based on initial needs assessment, planning, and implementation. In these sessions, patient engagement was achieved through discussions about the educational program, factors and risk factors for complications and symptom exacerbation, environmental factors and ways to address them, appropriate physical activity, creating or maintaining independence in daily living activities, and improving quality of life with the participation of the patient, active family member or caregiver, and nurse. Patients actively participated in discussions. Discussions between the patient, nurse, and active family member played an important role in this stage. Vital signs of all patients were checked, and appropriate action was taken by the doctor if there was a problem. They were familiarized with the medications they were taking, how to use them, and their side effects. If they had physical problems, they were advised to see a specialist in that field. At the end of these sessions, efforts were made to bring patients to an acceptable level of awareness, attitude, and performance for participation in the care and treatment process. During these sessions, an educational booklet prepared by studying reference books and approved by an expert was used (in three 45–60 min sessions at the patient's bedside during dialysis with the presence of an active family member).

*Engagement Stage*: These sessions (two sessions) aimed to continue the care program and engage patients. In the engagement stage, problems were addressed, behavior feedback was given to patients, and their level of participation was assessed. It was ensured that they were following what they learned in the previous stage. Nurses explained the follow-up care and recommendations, factors affecting non-compliance with treatment, necessary information, skills, and methods related to treatment compliance and its influencing factors, and discussed these topics with patients. Problems were reviewed, and previous educational actions and interventions were evaluated and revised by the researchers and the treatment team, providing necessary guidance to correct errors. Any questions or problems patients had were addressed in this stage. In all steps, education was provided simply and face-to-face by the researcher with the presence of an active family member (two 45–60 min sessions at the patient's bedside during dialysis).

*Evaluation Stage:* For the final evaluation of the model's implementation, the final stage, evaluation, was conducted. In the evaluation stage, the level of previous learning was assessed, and patients' attitudes were measured. Evaluation was done to follow up on treatment and recommended care. Compliance with dietary, medication, and exercise programs was checked. In this stage, patients' progress was regularly assessed, and feedback was given to them. This feedback included encouragement for small progress and providing new solutions for existing challenges. Patients were also encouraged to benefit from social and family support (one 45–60 min session at the patient's bedside during hemodialysis with the presence of an active family member).

Two months after the last educational session, the self-care and self-efficacy questionnaires were completed again in the same manner as before the study.

### 2.7 Data analysis method

Data analysis was performed using Stata software version 16. The decision to use parametric or non-parametric tests was based on the Kolmogorov-Smirnov test. For analyzing and comparing qualitative and quantitative data between the two groups, inferential statistical tests such as the Chi-square test, Fisher's exact test, independent *t*-test, and paired *t*-test were used. A significance level of less than 0.05 was considered.

### 2.8 Ethical considerations

This study was approved by the Ethics Committee of Hamadan University of Medical Sciences (Ethics Code: IR. UMSHA.REC.1402.498). Prior to the commencement of the study, the research objectives and the voluntary nature of participation were explained to the participants, and informed written consent was obtained from all patients. Participants were assured that their personal information would remain confidential throughout the data collection and reporting process and would not be disclosed to any individual or entity. During the study, integrity and honesty were maintained in the collection, recording, and analysis of data. Upon completion of the study, the educational booklet was also provided to the control group to ensure they received the same information as the intervention group.

## 3 Results

**Fig. 1:**
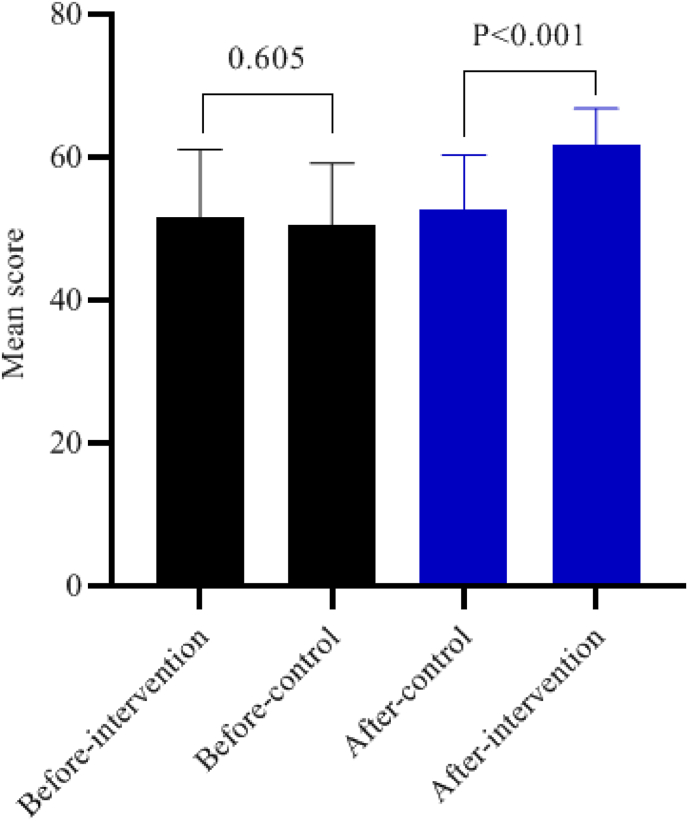
Comparison self-care between the two groups before and after the intervention.

A total of 37 participants were enrolled in each group. After the study process, considering the dropout of 4 participants in each group, 33 participants successfully completed the study (Fig. 1). The intervention and control groups did not show any statistically significant differences in terms of sex, marital status, occupation, education, number of dialysis sessions per week, age, duration of kidney problem, duration of dialysis, body mass index, duration of each dialysis session, and monthly household income (p-value >0.05). Therefore, the two groups were homogeneous regarding these variables (Table 1).

**Table 1:** Comparison of the demographic characteristics of the patients with heart failure in the intervention and control groups.

Demographic characteristics		Intervention group (N = 33)	Control group (N = 33)	P value
		N (%)	N (%)	
**Sex**	Female	16 (48.5)	15 (45.5)	0.805^a^
	Male	17 (51.5)	18 (54.5)	
**Marital status**	Married	28 (84.8)	28 (84.8)	0.311^b^
	Single	1 (3.0)	3 (9.2)	
	Spouse's death	4 (12.2)	1 (3.0)	
	Divorced	0 (0.0)	1 (3.0)	
**Occupation**	Household	14 (42.4)	14 (39.4)	0.777^b^
	Retired	11 (33.3)	9 (27.3)	
	Self-employed job	5 (15.2)	7 (21.2)	
	Employee	1 (3.0)	0 (0)	
	Unemployment	2 (6.1)	4 (12.1)	
**Education**	Illiterate	9 (27.3)	7 (21.2)	
	Below Diploma	11 (33.3)	16 (48.5)	0.207^b^
	Diploma	4 (8.3)	3 (6.1)	
	Associate Degree	0 (0.0)	3 (9.1)	
	Bachelor's Degree	2 (6.1)	0 (0.0)	
	Master's Degree and Above	1 (3.0)	1 (3.0)	
**Number of dialysis sessions per week**	2	5 (15.2)	2 (6.1)	0.372^b^
	3	25 (75.7)	30 (90.9)	
	4	3 (9.1)	1 (3.0)	
**Demographic characteristics**		**M±SD**	**M±SD**	P value^c^
**Age (years)**		59.03 ± 9.71	57.61 ± 10.95	0.578
**Duration of kidney problem (years)**		4.01 ± 1.97	4.19 ± 2.11	0.718
**Duration of dialysis (years)**		2.98 ± 2.31	3.02 ± 1.65	0.951
**Body mass index**		24.86 ± 5.93	24.90 ± 4.91	0.975
**Duration of each dialysis session (hours)**		3.70 ± 0.35	3.72 ± 0.38	0.761
**Monthly household income (million Toman)**		10.25 ± 4.72	10.15 ± 4.67	0.928

a Chi 2.

b Fisher Exact Test.

c Independent T Test.

**Fig. 2:**
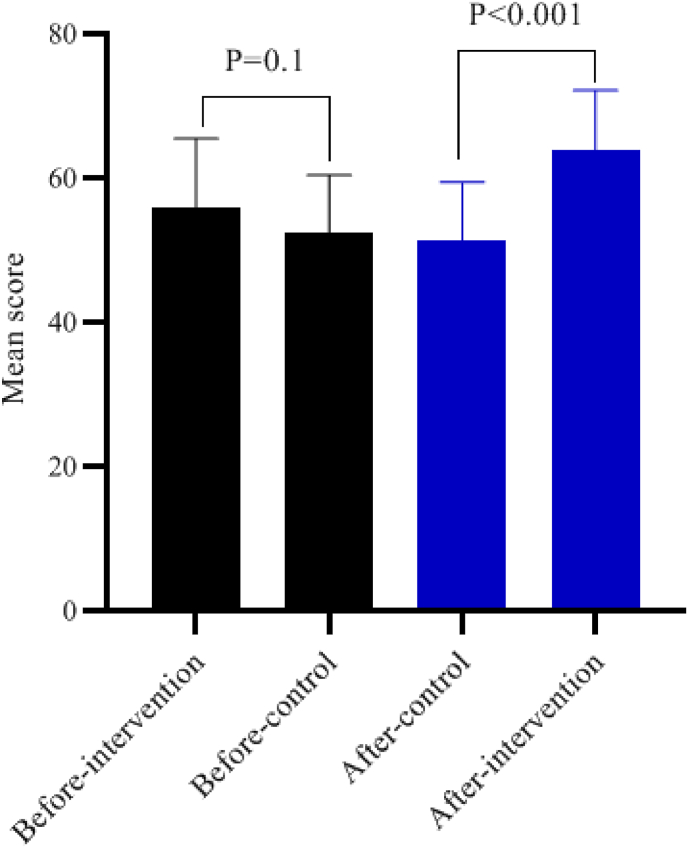
Comparison self-efficacy between the two groups before and after the intervention.

The results of the independent *t*-test indicated that there was no statistically significant difference in the mean scores of self-care and self-efficacy between the intervention and control groups before the intervention (P > 0.05). However, after the intervention, the mean scores of self-care and self-efficacy in the intervention group showed a significant increase compared to the control group (P < 0.001). Specifically, post-intervention self-care in the experimental group exhibited an effect size of 1.41 (95 % CI: 0.87–1.95), with a P-value of <0.001, indicating a substantial improvement. Similarly, self-efficacy in the experimental group had an effect size of 1.55 (95 % CI: 0.99–2.10), with a P-value of <0.001, demonstrating significant enhancement (Table 2. Fig. 1, Fig. 2).

**Table 2:** Comparison of study outcomes within and between the two groups before and after the intervention.

Variables	Time of evaluation	Experimental group (n = 33)	Control group (n = 33)	Effect size^c^ (95 % CI)	Statistic	P-value^a^
		M±SD	M±SD			
**Self-care (0-72)**	Before	51.73 ± 9.39	50.58 ± 8.57	0.13 (−0.36–0.61)	0.520	0.605
	After	61.79 ± 5.6	52.55 ± 7.76	1.41 (0.87–1.95)	5.730	<0.001
	statistic	−9.189	−3.090			
	p-value^b^	<0.001	0.004			
**Self-efficacy (17**–**85)**	Before	55.97 ± 9.52	52.33 ± 8.12	0.41 (−0.78–0.90)	1.669	0.1
	After	63.97 ± 8.20	51.36 ± 8.09	1.55 (0.99–2.10)	6.288	<0.001
	statistic	−7.720	0.434			
	p-value^b^	<0.001	0.161			

n = sample; SD = standard deviation, M = mean.

a Independent *t*-test،.

b Paired *t*-test.

c Cohens d.

Additionally, the paired *t*-test revealed that in the intervention group, the mean scores of self-care and self-efficacy significantly increased after the intervention compared to before (P < 0.001 for both). In the control group, the mean score of self-care also showed a significant increase after the intervention compared to before (P = 0.004); however, this difference was not statistically significant for the mean score of self-efficacy (P = 0.161) as shown in Table 2.

## 4 Discussion

This study aimed to determine the impact of a partnership care model on self-efficacy and self-care among hemodialysis patients attending Shahid Beheshti Educational and Medical Center in Hamadan. Overall, the findings indicate that the partnership care model can effectively enhance self-efficacy and self-care in hemodialysis patients.

The average self-care score in the control group showed a significant increase compared to the pre-study period, indicating that even routine care and education can lead to improvements in patient self-care. This improvement may be due to patients paying more attention to daily care and becoming more aware of the importance of self-care. Even with routine care, patients may benefit from the guidance and recommendations of nurses. These interactions can help increase patients' awareness and motivation for self-care. Another reason for this increase in self-care could be that hemodialysis patients often receive treatment in shared environments and may benefit from the experiences and recommendations of other patients. This exchange of information can help improve self-care. During this period, patients may also have access to educational resources through television, radio, and the internet, which can be effective in enhancing their self-care.

Overall, this comprehensive and multi-stage program significantly increased patients' awareness, motivation, and empowerment, leading to a markedly higher self-care score in the test group compared to the control group. The substantial effect size for self-care underscores the strong impact of the intervention. The accuracy of the program's implementation, along with the active involvement of patients, family members, and nurses, was pivotal. In the early stages of the intervention, emphasis was placed on motivating and preparing patients to engage in their own care, helping them comprehend the importance of self-care and find the motivation to adhere to their care plans. During the preparation stage, patients learned to plan their daily activities and prioritize tasks, reducing stress and enhancing their ability to manage their illness. They also became familiar with methods to mitigate physical and psychological complications, further improving their self-care. In the engagement stage, patients actively participated in discussions and decision-making processes, increasing their sense of responsibility and empowerment in managing their illness. The presence of a supportive nurse and an active family member in the sessions facilitated open communication and provided essential support, boosting patients' confidence in managing their condition. Finally, in the evaluation stage, patients' learning was assessed, and follow-ups ensured adherence to treatment plans. These follow-ups helped patients maintain continuous improvement and self-care practices [[34], [35], [36]].

This finding is consistent with the result that a self-management education program for patients with chronic kidney disease can improve self-care behaviors 4 and 8 weeks after the intervention [37]. In this context, self-efficacy can be seen as an intermediary factor that increases patients' self-care [38]. As a result, when the findings of this study were compared with the results of Lee et al.'s study on self-efficacy-focused interventions for patients with end-stage renal disease in the early stages, it showed that the intervention effectively improved self-management in patients with end-stage renal disease in the early stages after 3 months; this was the same result obtained in the present study [39]. Consistent with the present study, Ghavidel et al. showed that implementing a participatory model improved the quality of life of hemodialysis patients [40]. Hua et al. (2017) also showed that collaborative care, compared to usual care, improved self-care, quality of life, and cardiac function in patients with heart failure [26]. The similarities between our findings and those of previous studies suggest that self-efficacy and collaborative care models play a crucial role in enhancing self-care and overall well-being among patients with chronic conditions. The large effect sizes observed in this study indicate the strong impact of the intervention, similar to the outcomes reported in other studies. Possible reasons for these consistencies include the shared focus on empowering patients through education, active participation in care, and the involvement of supportive healthcare professionals and family members. These factors collectively contribute to improved self-management and health outcomes.

Results showed that routine care had no significant impact on the self-efficacy of hemodialysis patients. This may be due to insufficient focus on increasing patients' confidence and ability to care for themselves. It indicates that to improve self-efficacy, more specific and targeted nursing interventions and education are needed to help patients acquire the necessary skills and confidence to manage their illness.

After the intervention, the self-efficacy of the partnership care education group increased compared to the control group. The increase in self-efficacy in the test group was due to several key factors and psychological and educational mechanisms employed in the partnership care model. The substantial effect size for self-efficacy (1.55, 95 % CI: 0.99–2.10, p < 0.001) observed in the intervention group indicates a strong and meaningful impact of the intervention, underscoring the effectiveness of the partnership care model in enhancing self-efficacy among hemodialysis patients. Let's delve deeper into these factors: One of the most important factors in increasing self-efficacy is enhancing patients' knowledge and awareness about the disease and its management methods. When patients have sufficient information about their disease, they feel more in control of their situation, which leads to increased self-efficacy. Motivation and commitment to self-care are crucial factors in increasing self-efficacy. Other reasons include planning and organization, where in the preparation stage, patients learned how to plan their daily activities and prioritize their tasks. This planning helped reduce stress and increase their ability to manage the disease. Additionally, in the engagement stage, patients actively participated in discussions and decision-making [34]. This active participation increased their sense of responsibility and empowerment in managing the disease. The presence of a nurse and an active family member also helped patients feel more supported. Social support and active participation in decision-making are important factors in increasing self-efficacy [41]. Finally, in the evaluation stage, patients' learning was assessed, and necessary follow-ups were conducted to ensure adherence to treatment. These follow-ups helped patients continuously stay on the path of improvement and self-care. Continuous follow-up and evaluation help patients feel they are on the right track, which leads to increased self-efficacy [42].

Consistent with the present study, research results showed that using the collaborative care model in nursing can significantly improve patients' self-efficacy and meet their self-management needs [43]. Additionally, the results of Yan et al. (2021) align with the present study, showing that eight sessions of education based on the collaborative care model increased the self-efficacy scores of kidney transplant patients at 1, 2, and 3 months post-discharge [44]. In line with the present study, Borji and Motaghi showed that using the partnership care model in five educational sessions increased the self-efficacy of the elderly [45]. In the study by Sanaei et al. which examined the implementation of the collaborative care model on the self-efficacy and self-belief of family caregivers, their findings showed that implementing this model improved caregivers' self-efficacy and self-belief, leading to increased participation in providing effective care, consistent with the results of the present study [34]. The substantial effect size for self-efficacy observed in the present study underscores the strong impact of the collaborative care model. This finding aligns with previous studies that highlight the effectiveness of collaborative and partnership care models in enhancing self-efficacy and self-management among patients and caregivers. The consistency of these results can be attributed to shared key components in the interventions, such as patient education, active participation in care, and support from healthcare professionals and family members. These factors collectively contribute to improved self-management and health outcomes. The enhanced knowledge, motivation, and empowerment provided by these models help patients and caregivers feel more in control of their health, thereby increasing their self-efficacy.

Empowerment and strengthening are two fundamental concepts in the philosophy of collaborative care. Empowerment means providing opportunities for all members to demonstrate their current capacities and competencies and acquire new skills and abilities towards the goal(s). Strengthening, however, explains professional interactions with families to make them feel in control of their lives. As a result, families' abilities to perform health-promoting activities to achieve the goals are enhanced [[44], [45], [46]].

To effectively implement the findings of this study, it is crucial to design and execute partnership care model incorporating the stages of motivation, preparation, engagement, and evaluation. Such programs should encourage patients to actively engage in decision-making and planning concerning their self-care. Furthermore, it is imperative that nurses in these settings receive training on the application of this model.

### 4.1 Implications for practice

By delivering comprehensive education and offering social and motivational support, nurses can encourage active patient participation in self-care. Personalized care plans tailored to each patient's specific needs and conditions can be developed using this model, ensuring that individual requirements are addressed effectively. In the realm of nursing education, the study's results can inform the design and implementation of more effective educational programs for hemodialysis patients. These programs should encompass detailed information about the disease, its symptoms, complications, and management strategies. Active involvement of patients and their family members in educational sessions can boost their commitment and motivation to adhere to self-care practices. It is also recommended that this educational model be taught to nursing students, providing them with opportunities to practice and apply it in patient education. Additionally, training nurses on how to effectively implement this model can enhance their ability to deliver high-quality care.

By integrating these findings into clinical practice, healthcare managers, supervisors, and hospital administrators can develop more effective care and educational programs for hemodialysis patients. These programs should include stages of motivation, preparation, engagement, and evaluation, each carefully executed with the involvement of the patient, an active family member, and a nurse. Providing necessary resources and support can improve patients' quality of life and treatment outcomes. Moreover, offering continuing education workshops on the application of the partnership care model and recognizing nurses who employ this model can further enhance the quality of nursing care.

### 4.2 Strengths and weaknesses of the study

One of the strengths of this study was the use of the partnership care model and involving the patient and their family in patient care. Weaknesses include the short two-month follow-up period, conducting the intervention in a single medical center, and not examining other outcomes of collaborative care such as resilience, quality of life of the patient and their family. It would also have been better to compare this care model with other educational models.

## 5 Conclusion

The results of the study showed that using the partnership care model can significantly improve the self-care and self-efficacy of hemodialysis patients. The partnership care model, by providing comprehensive education, social and motivational support, and active patient participation, significantly improved the self-care and self-efficacy of hemodialysis patients. Therefore, it is recommended that hospital administrators and nurses use this indigenous, simple, efficient, and reliable model, which is compatible with Iranian culture and context, to educate and improve the health of hemodialysis patients to enhance their quality of life and treatment outcomes. In research, the study's findings can serve as a foundation for further investigations into the effects of the partnership care model on various aspects of patient health. Researchers can explore the impact of this model on different patient populations and compare its effectiveness with other care models. This can lead to the identification of best practices and the development of more refined and effective care strategies for patients with chronic conditions.

## CRediT authorship contribution statement

**Khodayar Oshvandi:** Writing – review & editing, Writing – original draft, Visualization, Validation, Formal analysis, Data curation, Conceptualization. **Hossein Moradi:** Writing – review & editing, Writing – original draft, Methodology, Investigation, Conceptualization. **Salman Khazaei:** Writing – review & editing, Writing – original draft, Supervision, Software, Resources, Funding acquisition, Formal analysis, Data curation. **Azim Azizi:** Writing – review & editing, Writing – original draft, Validation, Supervision, Methodology, Investigation, Conceptualization.

## Consent for publication

Not applicable.

## Funding

Financial support for this work was provided by the Office of the Vice-Chancellor for Research and Technology at 10.13039/501100004697Hamadan University of Medical Sciences, Hamadan, Iran (Grant Number 140208026411).

## Declaration of competing interest

The authors declare that they have no known competing financial interests or personal relationships that could have appeared to influence the work reported in this paper.

## Data Availability

Data will be made available on request.
